# Advancing Age‑Friendly Community Practice Through Global Learning: A Case Study of the Athens County–Slovenia Exchange for Health Equity

**DOI:** 10.5334/aogh.5048

**Published:** 2026-06-23

**Authors:** Gillian H. Ice, Amy Lipka, Ana Ramovš, Katja Bhatnagar, Alen Sajtl, Peter Beznec, Ruth Dudding

**Affiliations:** 1 Department of Social Medicine, Heritage College of Osteopathic Medicine, Ohio University, Athens, OH, USA; 2 Athens City County Health Department, Athens, OH, USA; 3 Anton Trstenjak Institute, Ljubljana, Slovenia; 4 The Centre for Health and Development Murska Sobota, Murska Sobota, Slovenia

**Keywords:** global health, health equity, aged, aging, program evaluation, age‑friendly communities, cross‑national exchange, older adults, global learning

## Abstract

*Background:* As populations age globally, communities are challenged to promote health equity and well-being for older adults. The World Health Organization’s (WHO’s) Global Network for Age-Friendly Cities and Communities provides a platform for sharing local innovations, while Global Learning for Health Equity (GL4HE) offers a framework for reciprocal, evidence-to-action learning across borders.

*Objective:* This case study examines a bilateral exchange between Athens County, Ohio, USA, and Slovenia to assess how short-term, immersive exchanges can stimulate innovation, clarify contextual factors, and build relationships that support age-friendly practice, following the GL4HE framework.

*Methods:* The project engaged a seven-member delegation from Athens County in Slovenia (January 2025) and a six-member Slovenian delegation in Athens County (May 2025). Delegations included representatives from local government, public health, community organizations, and academia. Itineraries combined structured site visits, informal reflection sessions, and cultural experiences. Documentation included agendas, minutes, reflections, evaluation, and message logs. Data were analyzed using a hybrid coding approach informed by the GL4HE framework and WHO Age-Friendly domains, with inductive coding to identify emergent themes.

*Results:* Five themes emerged: (1) inspiration sparks innovation, (2) comparative learning clarified context, (3) time for focus, (4) group reflection enhances learning, and (5) exchanges build relationships that inspire future collaboration. Participants reported both professional and personal transformation, with recognition that exchanges at the “explore” stage of GL4HE generate inspiration more than direct adoption.

*Conclusions:* Short-term exchanges are valuable vehicles for advancing age-friendly practice through global learning. By prioritizing a concentrated focus on a single issue (e.g., aging), reflection, and relationship building, exchanges provide a foundation for reciprocity and evidence-to-action transfer. Even without immediate adoption, such experiences can catalyze equity-oriented innovation and strengthen the relational infrastructure needed for sustained collaboration.

## Introduction

Across the world, populations are aging rapidly. Projections indicate that by 2030, one in every five people in the USA will be 65 or older, and globally, one in every six people will be over 60 [1]. Many older people face challenges such as ageism, limited resources, and mobility barriers [2–4] that impact well‑being. For this reason, aging is a growing health equity issue [2]. In recognition, the World Health Organization (WHO) established a Global Network for Age‑Friendly Cities and Communities in 2010 to connect communities that share a vision of “making their communities great places to grow old in” [5]. As populations age, communities around the world have developed innovative ways to engage older people that can improve health, access, and quality of life while also contributing to overall societal well‑being.

One approach to advancing these efforts is the Global Learning for Health Equity (GL4HE) framework. Defined as “the practice of engaging with, exchanging, and adapting health equity–promoting ideas and interventions between countries that fosters implementation benefits that are reciprocal and beneficial to both communities” [6], global learning for health equity emphasizes mutual knowledge sharing, local adaptation, and community engagement in addressing social determinants of health. The GL4HE framework recognizes 11 stages of global learning from “explore,” where communities examine models that may address a local equity issue, through to “impact” when outcomes are achieved [7].

This case study applies the lens of GL4HE to examine a partnership between Age‑Friendly Athens County, the Slovene Network of Age‑Friendly Cities and Communities, and the Centre for Health and Development Murska Sobota. The Athens team entered the GL4HE Framework at the “explore” stage to find a global partner who is advancing age‑friendly policies and practices. The explore phase involves identifying appropriate global partners, examining effective practices, and engaging in initial discussions to assess context fit and feasibility. At this stage, relationship building, exposure to diverse models, and critical reflection are emphasized. By documenting the exchange, identifying points of adaptation, and assessing the benefits for each community, we aim to demonstrate how age‑friendly initiatives can serve as a platform for reciprocal learning at this early stage that can promote health equity in aging populations.

While global learning and reciprocal innovation have been described in health equity and health systems research [6, 8, 9], their application to age‑friendly community practice remains underdeveloped in the USA. The WHO Age‑Friendly Cities and Communities emphasizes the exchange of experiences and practices across settings to spark innovation and improvement, encouraging communities to learn from one another while adapting to local context [10–14]. The Age‑Friendly Network promotes cross‑community learning; however, the process has not been widely conceptualized through a global learning or reciprocal innovation framework [11, 12], leaving a gap in understanding how global learning operates in this context.

## Case Study Description

### Age‑friendly athens county

The project was initiated in 2018 under the direction of Ohio University’s College of Health Sciences and Professions; an advisory council representing more than 80 community and individual partners was formed to develop and implement a survey aimed at collecting baseline data on the needs and priorities of Athens County residents. Athens County, Ohio, joined the American Association for Retired persons (AARP) Network of Age‑Friendly States and Communities in 2020 and the WHO Age‑Friendly Network in 2025 and is now guided by a coalition of public health, social services, higher education, community organizations, and Athens County residents [15]. The coalition reflects Athens County’s commitment to health equity and quality of life for older adults in a rural region and is supported by a full‑time coordinator with funding from the City of Athens, Athens County Commissioners, and local grants. Based on the survey results, transportation, housing, and community and health services were identified as the most pressing issues of the eight Age‑Friendly domains.

### The slovene network of age‑friendly cities and communities

Slovenia launched its Age‑Friendly Cities and Communities network in 2008, coordinated by the Anton Trstenjak Institute of Gerontology and Intergenerational Relations. The model is distinctive for its structured, systematic, and practical approach, engaging municipal age‑friendly coordinators and local action groups to ensure meaningful participation in each municipality. Its five‑year strategies adopted by each municipality focus on the development of three key areas: (1) long‑term care for frail and disabled populations, (2) active and healthy aging, and (3) intergenerational solidarity and education. Using a coordinated national–local approach, the network has expanded to more than 15 municipalities across Slovenia, with locally developed and managed programs addressing all eight age‑friendly domains. During the exchange, we focused particularly on preventing elder abuse, intergenerational programs, shared housing, transportation, and social participation.

### Rationale for exchange

Both Athens County and Slovenia participate in the WHO Global Network of Age‑Friendly Cities and Communities, yet operate on different scales of implementation. Athens County works through a community‑based coalition and is just beginning implementation, while Slovenia’s national network offers a coordinated system that has demonstrated longevity and serves as a global model of Age‑Friendly implementation. With some similarities in rural contexts, demographic aging, and resource constraints, Slovenia was a compelling model for Athens County to explore. At the same time, differences in healthcare systems, culture, and policy infrastructure provided an opportunity for reciprocal learning.

### Exchange design and framework

The exchange was supported by a grant through the University of Maryland Baltimore’s GL4HE, funded by the Robert Wood Johnson Foundation [7]. Athens County had previous experience adapting global solutions to the local context (e.g., Friendship Bench model from Zimbabwe) and sought to test global learning for the aging sector. The Athens City‑County Health Department, with the support of Ohio University, proposed a project to explore a variety of age‑supportive strategies to find solutions for equitable access to community engagement, health access, and quality of life for older persons [2, 4]. The project culminated in an exchange with two Slovenian partners, the Anton Trstenjak Institute of Gerontology and Intergenerational Relations and the Centre for Health and Development Murska Sobota, selected based on alignment with local priorities identified through the Age‑Friendly Athens County needs assessment. The delegation visits were approximately one week.

Prior to the exchange, the Athens team explored partnerships with Japan and Rwanda and began focusing on Slovenia after an introduction by AARP staff to the Anton Trstenjak Institute. Around the same time, the Athens team reconnected with the Centre for Health and Development Murska Sobota (CHD), a Slovenian organization they had worked with in the past. These two connections developed in parallel, through discussions about age‑friendly initiatives and eventually came together into a collaborative team that co‑designed the structure and focus of the exchange. Each side contributed ideas for site visits and discussion topics, ensuring the exchange reflected local contexts and cross‑cultural insights that could be adapted in other settings.

#### Slovenia visit (January 2025)

A seven‑member delegation was assembled based on the priority domains identified during the assessment process. This included representatives from the Athens City‑County Health Department, Ohio University, Hocking, Athens, Perry Community Action, and an Athens County Commissioner. Activities included discussions with mayors, site visits to intergenerational centers and volunteer transportation initiatives, exposure to Slovenia’s home health and health promotion programs, and inclusive social enterprises.

#### Athens county visit (May 2025)

A six‑member Slovenian delegation—including municipal leaders and staff from the national coordinating institute—spent one week in southeastern Ohio. Activities included meetings with the local age‑friendly coalition; visits to community‑led revitalization projects in small towns; observation of a rural food system and market innovations; exploration of aging‑in‑place support, transportation, and mobility initiatives; and engagement with inclusive social enterprises and university‑based programs linking aging, public health, and sustainability.

## Data and Analysis

The project was submitted to Ohio University’s institutional review board for ethics review and was deemed to be exempt from review. Shared documentation included agendas, meeting minutes, reflections, evaluations, message exchanges, and transcripts. These materials created a rich qualitative record of structured and unstructured learning moments. Data were analyzed through a hybrid AI‑assisted coding approach, combining elements from the GL4HE framework and WHO Age‑Friendly domains with themes emerging from participant reflections. Coding was conducted iteratively by the lead author with support from ChatGPT as an organizational and synthesis tool, alongside manual coding. Triangulation across data types, reflexive memos, and validation by co‑authors from both countries ensured rigor and balanced interpretation.

## Results

Using multiple data sources generated during two global learning delegation visits, including a total of 13 members of the delegation and 12 community host partners in Athens County, OH, we identified five themes.

### Inspiration sparks innovation

The exchange provided exposure to different practices that inspired participants to imagine possibilities at home. US delegates were impressed by Slovenia’s robust volunteer transportation services and caregiver support networks. One participant reflected, “The volunteer network and transportation services were particularly noteworthy, as they addressed transportation for errands and friendly visits—not just medical care” (US participant, Slovenia visit). These examples encouraged Athens leaders to consider how existing transportation services in Athens might meet other needs of older people than currently offered. Conversely, Slovenian leaders pointed to the Athens Village (a community nonprofit aging support cooperative) as an interesting grassroots model to improve access to home safety interventions and handyman services that could be adapted to their municipalities. One Slovenian delegate shared, “…we need to expand the home care assistance with smaller tasks, like handy man. … I think we need to help people with small changes in the adaptation of homes – for example in the bathroom to get holders, the removal of carpets in the house. I think as [a] municipality we could co‑finance or pay for them…” In both directions, the exchange provided inspiration for innovation. Following the exchange visits, local initiatives were developed or revised (see Table 1).

**Table 1:** Local impact following Athens–Slovenia exchange visits.

ATHENS COUNTY PRIORITY	EXCHANGE LEARNING	OUTCOME / LOCAL APPLICATION
Sustainable funding	Municipal support is key to sustaining age‑friendly work.	Athens: Strengthened the case for local public investment in Age‑Friendly Athens County.
Transportation	Volunteer and municipal transportation can support both mobility and social connection.	Athens: Informed local efforts to make existing transportation in Athens County easier for older adults to understand and use.
Housing	Housing should connect design, services, and community life.	Athens: Renewed local momentum around Community Reinvestment Area policy, with added emphasis on universal age‑friendly design in renovation and new construction.
Community and health services	Trusted local community members help connect older adults to support. “Handyman” and other support services can help older people live independently at home.	Athens: Reinforced local attention to Community Health Workers, caregiver support, health education, and safety programs. Slovenia: Exploring ways to adapt and implement these practices within the Slovenian context.
Intergenerational connection	Intergenerational work can be structured and intentional.	Athens and Slovenia: Led to collaboration with Mission of Generations to locally playtest the English version of its intergenerational game.
Rural community hubs	Existing gathering places are essential age‑friendly assets.	Athens: Validated a “start where people already gather” approach to local implementation.
Learning process	Reflection helped translate global learning into local action.	Athens: Reinforced motivation for local action and helped turn exchange insights into concrete planning and follow‑up. Slovenia: Integrated observed good practices into the training curriculum for new age‑friendly initiatives.

### Comparative learning clarifies context

Delegates consistently compared what they saw with their home contexts, increasing their understanding of barriers and facilitators in creating age‑friendly communities. US participants expressed surprise at the extent of volunteerism embedded in Slovenian age‑friendly municipalities. Following a municipality visit, one US delegate commented, “Our first exposure to how strong volunteer culture is among age‑friendly municipalities in Slovenia. Something we should definitely think more about how to implement here in Athens.” As the delegation learned more about volunteerism in Slovenia, several participants tied it back to their own challenges at home. For example, one participant noted, “Also universal was the challenge of maintaining staff and volunteers.” (US participant, Slovenia visit). Although Slovenian culture has a strong tradition of volunteerism, challenges related to volunteer management were shared across communities. Slovenians were impressed by how Athens County communities leveraged limited resources to build creative programs, such as the Glouster Depot Project, which was started by a group of women who transformed a vacant train depot into a quilting workshop. They also visited the Chesterhill Produce Auction, which connects Amish farmers to the wider public through produce sales. As one Slovenian delegate reflected, “I was impressed how you tried to put community together. In Amesville, in Glouster… Athens Village is a project which I really want to do in the future.” In seeing contrasts, participants were able to see challenges not as unique but as a part of broader patterns, encouraging new thinking about feasibility and adaptation to home contexts.

### Time for focus

Although not part of the original lens, the theme of concentrated time for reflection emerged through the iterative coding process. Participants from both countries emphasized the value of spending several days, exclusively focused on aging. One Slovenian mayor reflected: “For me as a mayor it was very important that I had five days’ time to think about older people. When you have a lot of functions in your job description, you hardly think just about older people.” Time away from competing demands resonated with other exchange participants, allowing them to synthesize learning, and renew their commitment to aging work. For US delegates newer to the field, the exchange provided an opportunity to adopt what one described as an “age‑friendly lens.” One participant reflected, “So I am probably the least involved in age friendly activities, or I was until now. I’m signed up, I guess. And so I feel like for me, it’s just given me a new lens of how to see everything. And I think the intergenerational piece was really what resonated the most with me….” Concentrated time thus served as a mechanism for professional development and mindset shift, even before concrete program changes are planned.

### Group reflection enhances learning

For both delegations, learning occurred not only through formal site visits but also in the informal discussions within delegations and with hosts during meals, car rides, and downtime. As one US participant noted, “The conversations that continued in the car and over meals were equally enriching.” These reflections allowed participants to process the day’s activities, compare interpretations, and reinforce key insights. Slovenian participants similarly highlighted the value of evening discussions to digest complex ideas and brainstorm applications. As one participant noted, “I generally enjoyed all of our companies… I was learning even how politics work. … And the second thing… it made us think, I think all of us from Institute or municipalities, …here we could have a little bit more time to think…” (Slovenian reflection, USA visit). In addition, delegates from both sides were able to gain more insight into context through informal discussions with hosts. This less scripted form of peer‑to‑peer learning deepened the impact of the exchange, creating shared experiences, and reinforcing motivation across the group.

### Exchanges build relationships that inspire future collaboration

A critical part of the GL4HE model is reciprocity and mutual trust. Perhaps the most important outcome of this exchange was strengthening relations and trust between the two delegations. Participants in both delegations repeatedly expressed gratitude for planning and hospitality, often voicing hope for future collaboration. “I hope, certainly hope, we will collaborate in future…this could be the start of a new, beautiful collaboration, if not friendship.” (Slovenian delegation, US visit). Beyond technical inspiration, participants concluded their exchange visit with personal bonds, pride in their communities, and a shared story that they can carry forward. Having a relational foundation is critical to establishing a long‑term, sustainable collaboration. While short visits provide more superficial learning, the exchanges create the conditions for reciprocity and translocation transfer that are central to the GL4HE framework.

## Discussion

GL4HE framework emphasizes reciprocal, evidence‑to‑action learning across borders as a pathway to advancing health equity [16]. The WHO Global Network for Age‑Friendly Cities and Communities was established to inspire change, connect communities, and support innovative, evidence‑based solutions to improve the lives of older people [5, 11, 14]. In this sense, the network can function as a vehicle for GL4HE. This case study demonstrates that short, immersive exchanges during the “explore” phase of the GL4HE framework can play a critical role in the early inspiration phase, stimulating ideas, sparking innovation, and laying the relational foundation for deeper reciprocity critical to partnership. In this case, participants engaged in inspiration and recognition of possibilities more than direct adaptation, which is a necessary step to begin the global learning cycle described by Shin et al. [16].

According to Buffel et al. [17], three benefits of comparative study of age‑friendly initiatives are identified—(1) knowledge generation of diverse methods of assessment, (2) improving understanding of barriers and factors for success of interventions, and (3) inspire mutual learning to better serve older persons. In this case, in‑person exchange of two WHO Age‑Friendly Network members allowed for global learning by providing uninterrupted days to focus on aging and including participants who had limited or no experience with an “age‑friendly lens.” Further, the site visits and associated discussion allowed for those actively in the aging service sector to examine services in a new light. The benefits of comparative study and in‑person exchange were realized in this project in concrete ways (Table 1). In both locations, current activities were revised and new ones were inspired. As such, this exchange demonstrated how the GL4HE “explore” phase helps participants consider whether ideas observed in one context could be adapted meaningfully in another [7, 16].

The importance of formal and informal reflection is a recognized best practice for global learning [18–20]. Impactful global learning facilitates a deeper understanding not only of the intervention but also allows learners to assess transferability through appreciation of context [8, 9, 21]. Rather than direct transfer, exchange visits provided analytic clarity about why certain strategies thrive in one context but require adaptation in another. The trust and camaraderie that develops can form a foundational structure for relationships that can support meaningful collaboration and eventual evidence‑to‑action transfer consistent with GL4HE principles and comparative learning theory [6, 22, 23]. In this case, the exchange created an opportunity to establish the beginnings of reciprocity that may yield more substantive joint projects in the future.

The exchange laid the foundation for future collaboration between the Anton Trstenjak Institute and Ohio University, with the goal of exploring research collaboration. While a Memorandum of Understanding is in place, there are not yet specific plans for joint projects. In line with the evidence‑to‑action framework, the exchange served to spark community dialogs around possible adaptation of interventions/services in Athens County and Slovenian municipalities. The exchange helped participants shift their thinking about age‑friendly approaches. For example, in Log Dragomer, a multiuse path was highlighted as an age‑friendly project. The multiuse path in Athens County was built for recreation, yet it serves as an age‑friendly asset that promotes safety, connection, and active living. It shifted the perspective from asking “what new things are needed” to “how can we better value and connect what we already have?” Similarly, the exchange informed local housing policy discussions, including work by an Athens County Commissioner to incorporate age‑friendly principles into a Community Reinvestment Area policy. We now think of this as aging in every policy and process. These shifting perspectives exemplify the value of immersive comparative study, including generation of knowledge, greater understanding of success factors and barriers to programs that support equity, and foster new ways to improve the lives of underserved populations [7, 13, 16, 17, 24, 25].

## Conclusions and Recommendations

At the explore stage of the GL4HE framework, the Athens County–Slovenia exchange demonstrates that immersive experiences can stimulate innovation, clarify barriers and facilitators to solutions, and provide a space for reflection and peer learning. To maximize impact, we recommend that programs prioritize concentrated focus on a single issue (e.g., aging) and allow practitioners to step away from daily responsibilities. When reflection is built into a formal program, it maximizes learning; reflection and casual discussion during downtime builds trust. Finally, we recommend that practitioners embrace inspiration as a legitimate outcome, recognizing that the first stage of GL4HE is sparking ideas and commitment. Together, these findings affirm that even without immediate evidence‑to‑action, global learning exchanges are valuable vehicles for stimulating equity‑oriented innovation in age‑friendly practice. Experiences like these broaden our sense of community among Age‑Friendly practitioners contributing to a true global learning community of practice.
